# Design and Analysis of a Two-Degree-of-Freedom Inertial Piezoelectric Platform

**DOI:** 10.3390/ma18214995

**Published:** 2025-10-31

**Authors:** Qingbing Chang, Yicheng Xu, Xian Deng, Xuan Liu, Liangkuan Zhu, Jian Li, Yingxiang Liu

**Affiliations:** 1 School of Mechatronics Engineering, Northeast Forestry University, Harbin 150040, China; x2429028951@163.com (Y.X.); 19110757586@163.com (X.D.); lx2249110419@163.com (X.L.); zhulk@nefu.edu.cn (L.Z.); 2 Heilongjiang Provincial Key Laboratory of Forestry Intelligent Equipment, Harbin 150040, China; 3 State Key Laboratory of Robotics and Systems, Harbin Institute of Technology, Harbin 150001, China

**Keywords:** piezoelectric platform, inertial drive, cross-scale motion, flexure hinge mechanism

## Abstract

Leaf stomatal density directly regulates the rates of gas exchange and water loss and is a core indicator of plants’ water-retention capacity and drought adaptability. Because detecting leaves over a macroscopic range requires large-stroke motion, whereas accurate identification of stomata demands high-precision positioning, the operational platform for stomatal-density detection faces the dual challenge of large strokes and high resolution. This paper proposes a novel two-degree-of-freedom (2-DOF) cross-scale piezoelectric platform that employs a new three-degree-of-freedom (3-DOF) piezoelectric stator to address the backlash issue in inertial drive and combines it with finite-element simulation for verification. The prototype of the 2-DOF cross-scale piezoelectric positioning platform is developed, and a series of experiments are conducted to evaluate its performance. The experimental results show a motion range of 15 mm × 15 mm; the displacement backlash rates in the X and Y directions range from 0% to 9.84% and 0% to 28.42%, respectively; and the displacement resolutions reach 11.39 nm and 13.61 nm, respectively. In addition, an application experiment on leaf stomatal-density detection is carried out on the developed 2-DOF platform, demonstrating its potential for botanical micro-detection.

## 1. Introduction

With the development of micro-/nano-technology, micro-/nano-manipulation platforms play an increasingly important role in fields such as biomedical engineering, piezoelectric ceramics remain the workhorse for high-resolution actuators [1,2], micro-/nano-fabrication, and biological detection. Representative examples include plant stomatal detection mechanisms [3,4], microneedle aspiration-based cell extraction mechanisms [5,6], microfluidic chip-based cell sorting mechanisms [7], and nanotweezer grasping-based cell acquisition mechanisms [8]. Taking leaf stomatal-density detection as an example, two-degree-of-freedom positioning platforms are required to achieve precise control of leaf motion trajectories and accurate positioning [9,10]. Traditional positioning platforms driven by linear motors suffer from drawbacks such as low precision, electromagnetic interference, and structural complexity [11,12]. In contrast, piezoelectric actuators offer advantages including high resolution, immunity to electromagnetic interference, fast response, and a large stroke range [13,14,15,16]; therefore, they are adopted as the driving unit to construct precision positioning platforms.

Research on piezoelectric positioning platforms has been conducted for many years [17,18,19]. For instance, Wang et al. proposed a stick–slip positioning platform resistant to load variations [20]; by adopting a form-closed cam, the driving unit is separated from the moving unit to eliminate the influence of external loads. This platform has a stroke of 2 mm and an incremental step size ranging from 30 nm to 2.3 μm; however, its positioning accuracy is significantly affected by the backlash between the cam and the transmission mechanism, and it is also limited by a short stroke. Zhang et al. designed a novel X–Y nano-positioning platform [21], which features a displacement amplification ratio of 8.9, a resolution of 20 nm, and a motion range of 127 μm, but its mechanical stiffness is insufficient. Abedi et al. developed a two-degree-of-freedom (2-DOF) precision positioning platform based on S-shaped flexure hinges [22]; the platform has overall dimensions of 180 mm × 180 mm and provides a displacement of 150 μm along the X and Y directions, yet its structure is overly complex. In general, piezoelectric positioning platforms usually require strokes ranging from tens to hundreds of micrometers, while even larger strokes are demanded in complex environments. Therefore, inertially driven piezoelectric platforms are adopted to address this issue [23,24].

Inertial drive can achieve a large working range through cumulative stepping motion; however, inertially driven piezoelectric platforms suffer from significant displacement backlash. Displacement backlash not only reduces driving efficiency and positioning accuracy but also causes adverse wear on the mover, thereby shortening service life and reducing the reliability of piezoelectric actuators [25,26]. To address this phenomenon, researchers have conducted a series of studies [27,28,29]. For example, Tang et al. proposed a sequential control method using a single-chip microcontroller to suppress displacement backlash [30]. Comparative experiments between the microcontroller-based method and operation under the traditional stick–slip principle showed that backward motion could be significantly suppressed. Nevertheless, this method realizes motion only in a single degree of freedom and is not applicable in complex environments. Duan et al. proposed a stick–slip piezoelectric actuator based on a V-shaped driving mechanism to achieve a larger lateral displacement [31]. Backward displacement was reduced by adjusting the driving waveform, but the suppression effect was unsatisfactory, with a backlash rate of 86%. Ling et al. designed a stick–slip piezoelectric actuator based on the asymmetric-stiffness principle: in terms of structural design, a dual-driving-foot configuration and an asymmetric-stiffness compliant mechanism were adopted; in principle, the phase difference generated by the stiffness difference between the two driving feet was used to compensate for the backlash motion, resulting in a backlash rate of 35% [32].

In summary, research on piezoelectric positioning platforms has made certain progress, but existing achievements still have clear limitations: some platforms are greatly affected by backlash errors, have short strokes, or suffer from insufficient stiffness. To meet the demand for larger strokes in complex environments, the inertial-drive method has been applied to piezoelectric platforms; however, the resulting displacement backlash reduces driving efficiency and positioning accuracy, exacerbates mover wear, and impairs the service life and reliability of the actuators [33,34,35].

To address the aforementioned issues, a novel cross-scale piezoelectric positioning platform is proposed. This platform takes a three-degree-of-freedom piezoelectric stator as its core component, and, by virtue of this unique stator structure, it can resolve the backlash phenomenon of inertial drive. The remainder of this paper is organized as follows. Section 2 provides a detailed description of the working principle of the two-degree-of-freedom piezoelectric platform. Section 3 introduces the theoretical analysis of static modeling, and finite-element simulation of the piezoelectric platform is conducted using Ansys 17.0. In Section 4, the prototype is constructed, and experiments are carried out to evaluate the designed positioning platform. Finally, Section 5 presents a summary of this research.

## 2. Structural Design and Working Principle of a Two-Degree-of-Freedom Inertial Piezoelectric Platform

### 2.1. Platform Structure

**Figure 1:**
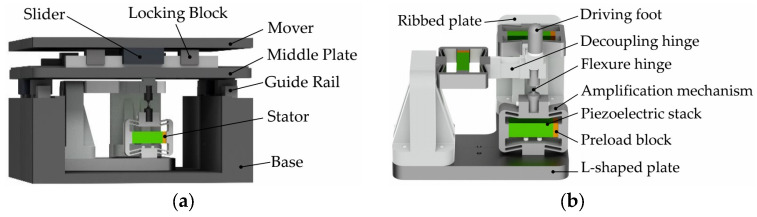
Two-degree-of-freedom piezoelectric platform structure: (**a**) two-degree-of-freedom inertial-driven piezoelectric platform; (**b**) three-degree-of-freedom piezoelectric stator structure.

The structure of the proposed two-degree-of-freedom piezoelectric positioning platform is shown in Figure 1a. It consists of 1 flat mover, 4 linear guide rails, 4 sliders, 8 limit blocks, 1 base, 1 connecting plate, 1 three-degree-of-freedom (3-DOF) piezoelectric stator, and several bolts. Among these components, the 3-DOF piezoelectric stator is fixedly mounted on the base. The moving platform, connecting plate, and base are slidingly connected in sequence, with the sliding directions of the connecting plate and the moving platform both horizontal and mutually perpendicular. The top of the 3-DOF piezoelectric stator is in clearance fit with the moving platform [36]. The connecting plate adopts a stepped structure, which ensures the guiding accuracy of the guide rails. Since the theoretical stroke of inertial drive is infinite, the motion range of the platform depends on the positions of the guide-rail limit blocks [37,38]; a motion stroke of 15 mm × 15 mm is adopted in this study. The overall dimensions of the 2-DOF piezoelectric positioning platform are 122.5 × 122.5 × 58 mm^3^.

The detailed structure of the 3-DOF piezoelectric stator is shown in Figure 1b. It consists of 3 piezoelectric stacks, 3 rhombic amplification mechanisms, 1 driving foot, 1 decoupling hinge, 2 lateral fixed seats, and 1 circular flexure hinge. Each driving mechanism of the 3-DOF stator is composed of one piezoelectric stack and one rhombic amplification mechanism. Among these three driving mechanisms, two are responsible for motion in two degrees of freedom, while the third is used to suppress displacement backlash. The intermediate flexure hinge and the circular flexure hinge enable motion of the platform’s orthogonal structure and achieve spatial decoupling. The driving foot is connected to the intermediate flexure hinge via a threaded connection, and adjustment shims of different thicknesses can be used to set the clearance between the driving foot and the platform. The piezoelectric stacks achieve displacement amplification through the rhombic amplification mechanisms; for the lateral stacks, these mechanisms also serve a direction-changing function.

### 2.2. Platform Working Principle

**Figure 2:**
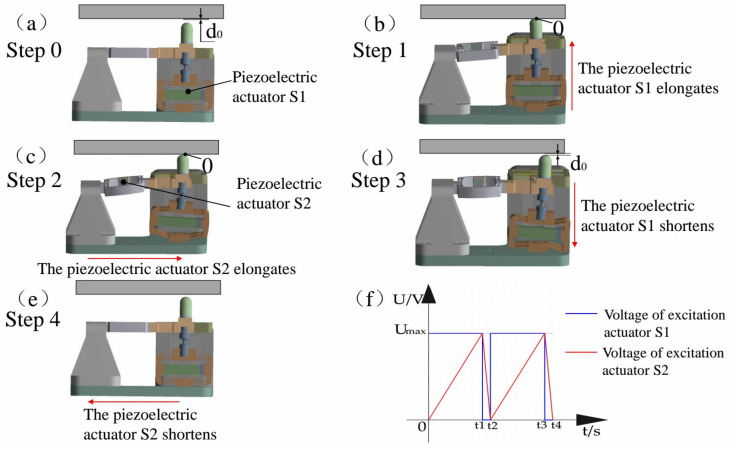
Design of motion generation strategy for Inertial piezoelectric platform: (**a**–**e**) analysis of motion generation process for inertial piezoelectric positioning platform; (**f**) excitation signal of piezoelectric actuator.

The 2-DOF piezoelectric positioning platform applies control voltages to the piezoelectric stacks and drives them with sawtooth-wave signals, enabling micrometer-level stepping motion and nanometer-level positioning accuracy. The driving principle is illustrated in Figure 2.

Step 0 (initial state): Before power is applied, as shown in Figure 2a, all piezoelectric actuators maintain their nominal lengths. At this point, there is a small clearance gap between the driving foot and the platform, so the preload force is zero.

Step 1: As shown in Figure 2b, the lower piezoelectric actuator S1 is driven by the blue voltage during the interval from 0 to t1, as depicted in Figure 2f. The clearance between the driving foot and the platform closes, and a preload force is established.

Step 2: As shown in Figure 2c, the lateral piezoelectric actuator S2 is driven by the red voltage during the interval from 0 to t1, as shown in Figure 2f. With sufficient preload, the lateral actuator S2 drives the platform to move.

Step 3: As shown in Figure 2d, after the lateral actuator S2 extends to its maximum displacement, it begins to retract. At this time, the lower actuator S1 is driven by the blue voltage during the interval from t1 to t2, as illustrated in Figure 2f. The lower actuator S1 contracts, moving the driving foot downward, and the clearance between the platform and the driving foot reappears.

Step 4: As shown in Figure 2e, the preload force between the platform and the driving foot is zero. The lateral actuator S2 is driven by the red voltage during the interval from t1 to t2, as presented in Figure 2f. The lateral actuator contracts and the driving foot retracts, while the platform remains stationary, returning to the state of Step 0.

By cyclically executing Steps 1 through 4, the proposed inertial piezoelectric platform effectively addresses the retraction problem inherent to inertial drive. The working principle in the other degree of freedom is similar, and the excitation signals of the piezoelectric actuators are shown in Figure 2f.

## 3. Theoretical Modeling and Simulation of the Driving Stators

### 3.1. The Establishment of the Output Displacement Model

**Figure 3:**
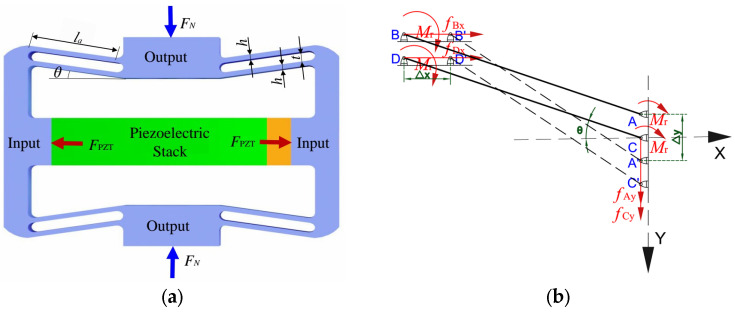
Rhombic flexible mechanism: (**a**) schematic diagram of the rhombic flexible mechanism; (**b**) force analysis of the rhombic flexible mechanism.

The design of piezoelectric positioning platforms typically imposes preconditions on flexure hinges, such as requiring the material’s allowable stress to exceed the internal stress, keeping the elastic recovery force below the maximum driving force, and ensuring strong anti-interference capability [39]. As shown in Figure 3a, the rhombic flexure amplification mechanism has a hypotenuse angle of 10 degrees, a hypotenuse thickness of 0.50 mm, and overall dimensions of 36 mm × 22 mm × 5 mm. The piezoelectric ceramic stack is a laminated actuator (PZT-4, Harbin Core Tomorrow Technology Co., Ltd., Harbin, China), with dimensions of 5 mm × 5 mm × 18 mm. A ceramic gasket measuring 5 mm × 5 mm × 5 mm is bonded to one end of the stack. As shown in Figure 3b, let F_PZT_ and F_N_ denote the output force of the piezoelectric stack and the external load, respectively. When a positive voltage is applied, the displacement amplification mechanism elongates in the horizontal direction by two times delta x and simultaneously contracts in the vertical direction by two times delta y. Owing to symmetry, one quarter of the rhombic flexure displacement amplification mechanism is taken for mechanical analysis, leading to the relations *F_PZT_ =* 4*f_x_* and *F_N_ =* 4*f_y_*. As illustrated in Figure 3b, the flexible dual-arm mechanism comprises flexible arm AB and flexible arm CD. Because these two arms have identical mechanical boundary conditions and geometric parameters, the analysis can be simplified by using arm AB as a representative.

Considering the fixed boundary conditions of arm AB, points A and B have equal rotation angles; point A translates horizontally, and point B translates vertically. Constrained by points A and B, arm AB is subjected to a bending moment equal to two times *M_r_*. Based on force and moment balance, the corresponding equilibrium relations can be obtained.

(1) $$f_{Ax}=f_{Bx}=f_{x}=f_{PZT}/4$$

(2) $$f_{Ay}=f_{By}=f_{y}=f_{N}/4$$

(3) $$2M_{r}=f_{x}l_{a}\sin\theta +f_{y}l_{a}\cos\theta$$

Here, *l_a_* and *θ* represent geometric parameters of the rhombic amplification mechanism, as shown in Figure 2a; *f_x_* and *M_r_* denote the force and the torque applied to the rhombic amplification mechanism, respectively; A, B, C, and D all denote the positions of the hinge mechanism endpoints before deformation; A’, B’, C’ and D’ all denote the positions of the hinge mechanism endpoints after deformation.

Force analysis is performed on the simplified flexible arm AB. Along its axis, arm AB is subjected to a tensile force; perpendicular to its axis, it is subjected to a transverse couple. In addition, the constraints at points A and B introduce a resultant moment of 2*M_r_* acting on AB, which causes the arm to bend during motion and thereby produces an amplified displacement output. Based on force and torque equilibrium, the expressions for the internal force and torque at a distance *u* from point A are as follows:

(4) $$\left.\begin{array}{l} f_{N}=f_{x}\cos\theta -f_{y}\sin\theta \\ M=M_{r}-(f_{x}u\sin\theta +f_{y}u\cos\theta ) \end{array}\right\}$$

Based on the Euler–Bernoulli beam theory, the total strain energy of the flexible arm is the sum of the tensile and bending strain energies, i.e., the tensile strain energy plus the bending strain energy.

(5) $$V_{\varepsilon }=V_{\varepsilon 1}+V_{\varepsilon 2}$$

According to Castigliano’s Second Theorem, the displacement of the mechanism along the x-direction.

(6) $$\begin{array}{cc} △x & =\frac{\partial V_{\varepsilon }}{\partial f_{x}}=\frac{f_{N}l_{a}}{EA}\frac{\partial f_{N}}{\partial f_{x}}+\int_{0}^{l_{a}}\frac{M}{EI}\frac{\partial M}{\partial f_{x}}du\\ & =\frac{l_{a}}{EA}(f_{x}\cos\theta -f_{y}\sin\theta )\cos\theta +\frac{l_{a}^{3}}{12EI}(f_{x}\sin\theta +f_{y}\cos\theta )\sin\theta \end{array}$$

The displacement of the mechanism along the y-direction is given by:

(7) $$\begin{array}{cc} △y & =\frac{\partial V_{\varepsilon }}{\partial f_{y}}=\frac{f_{N}l_{a}}{EA}\frac{\partial f_{N}}{\partial f_{y}}+\int_{0}^{l_{a}}\frac{M}{EI}\frac{\partial M}{\partial f_{y}}du\\ & =\frac{l_{a}}{EA}(f_{x}\cos\theta -f_{y}\sin\theta )\sin\theta +\frac{l_{a}^{3}}{12EI}(f_{x}\sin\theta +f_{y}\cos\theta )\cos\theta \end{array}$$

Here, *EA* and *EI* denote the tensile rigidity and the flexural rigidity of flexible arm AB, respectively. Based on Equations (6) and (7), the displacement amplification ratio of the amplification mechanism under the no-load condition (*f_y_* = 0) is given by:

(8) $$A_{d}=\frac{△y}{△x}=\frac{(l_{a}^{2}-h^{2})\sin\theta \cos\theta }{l_{a}^{2}6\sin^{2}\theta +h^{2}\cos^{2}\theta }$$

where *h* represents the geometric parameter of the rhombic amplification mechanism, as shown in Figure 3a.

**Figure 4:**
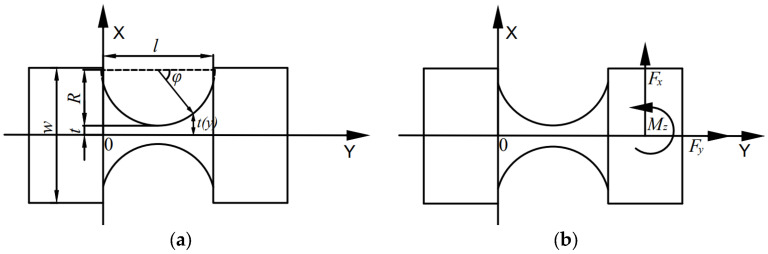
Straight circular flexure hinge: (**a**) schematic diagram of the straight circular flexure hinge; (**b**) force analysis diagram of the straight circular flexure hinge.

The structure of the single-sided straight-circular flexure hinge is shown in Figure 4. As depicted in Figure 4a, *R* is the cutting radius of the flexure hinge, *t* is the minimum thickness, and *w* is the hinge width. The loading condition is shown in Figure 4b: the left end of the flexure hinge is fixed, while the right end is free and subjected to a horizontal force *F*, a vertical force *F_y_*, and a couple *M*.

Based on linear elasticity and the small-deformation assumption, the displacement–load relation for the free end of the single-sided straight–circular flexure hinge under applied loading is given by:

(9) $$△=\left[\begin{array}{c} {△}_{x}\\ {△}_{y}\\ \theta_{z} \end{array}\right]=\left[\begin{array}{ccc} C_{11} & C_{12} & 0\\ 0 & C_{22} & 0\\ 0 & C_{32} & C_{33} \end{array}\right]\left[\begin{array}{c} F_{x}\\ F_{y}\\ M_{z} \end{array}\right]=CF$$

In the equation, Δ = (Δ*_x_* Δ*_y_ θ_z_*)^T^ denotes the displacement vector at the free end of the single-sided straight–circular flexure hinge, where Δ*_x_* and Δ*_y_* are the linear displacements along the X and Y directions and *θ_z_* is the rotation about the Z-axis; *F* = (*F_x_ F_y_ M_z_*)^T^ denotes the load vector applied at the free end; and *C*∈*R*^3×3^ is the flexibility matrix of the single-sided straight–circular flexure hinge.

When the free end of a single-sided straight–circular flexure hinge is subjected to the combined action of a horizontal force *F_x_*, a vertical force *F_y_*, and a couple moment *F_r_*, its total strain energy comprises the axial tensile strain energy and the bending strain energy.

(10) $$V_{\varepsilon 3}=\int_{0}^{l}\frac{F_{y}^{2}\left(y\right)}{2EA\left(y\right)}dx+\int_{0}^{l}\frac{\left[M_{z}+F_{x}{\left(l-y\right)}^{2}\right]}{2EI\left(y\right)}dy$$

In this equation, *V_ε_*_3_ denotes the strain energy of the straight–circular flexure hinge; *E* is the material’s elastic modulus in tension and compression; *A*(*y*) is the cross-sectional area of the hinge; and *I*(y) is the second moment of area (area moment of inertia) of the hinge cross-section.

The thickness of the flexure hinge at an arbitrary position *x* is given by:

(11) t(y)=R+t−Rsinφ,φ∈(0,π)

The relationship between an arbitrary x and the angle φ is given by:

(12) y=R−Rcosφ,dx=Rsinφdφ

The area of an arbitrary cross-section is given by:

(13) A(y)=wt(y)

The second moment of area of an arbitrary cross-section is given by:

(14) $$I(y)=\frac{wt^{3}(y)}{12}$$

Under the action of *F_x_* and *M_r_*, the linear displacement produced by the single-sided straight–circular flexure hinge along the *Δx* direction is given by:

(15) $$\begin{array}{cc} △x & {=C}_{11}F_{X}+C_{12}M_{Z}=\frac{\partial V_{\varepsilon 3}}{\partial Fy}=\int_{0}^{l}\frac{\left[F_{x}(l-y)+M_{z}](l-y\right)}{EI(y)}\\ & =\frac{12}{Et(y)}[\int_{0}^{\pi }\frac{F_{x}{\left(R+R\cos\varphi \right)}^{2}R\sin\varphi }{{\left(R+t-R\sin\varphi \right)}^{3}}d\varphi +\int_{0}^{\pi }\frac{M_{z}{\left(R+R\cos\varphi \right)}^{2}R\sin\varphi }{{\left(R+t-R\sin\varphi \right)}^{3}}d\varphi ] \end{array}$$

From Equation (14), when the single-sided straight–circular flexure hinge is subjected to the force *F_x_* along the x direction, the linear deformation flexibility *C*_11_ of the free end in the x direction is given by:

(16) $$\begin{array}{cc} C_{11}= & \frac{6}{Ew}\left\{\frac{1}{{\left(N^{2}-1\right)}^{\frac{9}{2}}}\left[2N^{3}\left(N^{4}-2N^{2}+1\right)\left(2N^{2}-5\right)\times \left(\arctan\frac{N-1}{\sqrt{N^{2}-1}}+\arctan\frac{1}{\sqrt{N^{2}-1}}\right)\right.\right.\\ & -{\left(N^{2}-1\right)}^{\frac{5}{2}}\left(\pi N^{4}+2N^{3}-2\pi N^{2}-N-\pi -4\right) \end{array}$$

In the equation: N=2t/l+1.

When the single-sided straight–circular flexure hinge is subjected to the couple *M_z_*, the linear deformation flexibility *C*_12_ of the free end in the x direction is given by:

(17) $$\begin{array}{cc} C_{12}= & \frac{12}{Ewl}\left\{\frac{1}{N{\left(N^{2}-1\right)}^{\frac{5}{2}}}\left[6N^{2}\left(\arctan\frac{N-1}{\sqrt{N^{2}-1}}+\arctan\frac{1}{\sqrt{N^{2}-1}}\right)\right.\right.\\ & \left.\sqrt{N^{2}-1}\left(3N^{2}+2N+2\right)\right]+\frac{1}{{\left(N^{2}-1\right)}^{\frac{5}{2}}}\\ & \left.\left(N\sqrt{N^{2}-1}-2\sqrt{N^{2}-1}+6\arctan\frac{N+1}{\sqrt{N^{2}-1}}\right)\right\}\; \end{array}$$

One end of the rhombic amplification mechanism is fixed and the other is free. The piezoelectric stack delivers a force of 2000 N at 150 V. Owing to the platform’s symmetry in the X and Y directions, only the x-direction displacement is analyzed. From Equation (14), the X-direction displacement ΔX is determined by the horizontal force and the X-direction linear flexibility *C*_11_. The output force *F_x_* of the rhombic amplification mechanism is calculated as 553.75 N using Equations (1)–(8). Then N and C_11_ are obtained from Equations (9)–(17). Finally, the X-direction displacement is 10.12 μm.

### 3.2. Finite Element Analysis of the Stator

The Finite Element Method (FEM) was employed to analyze the static and dynamic characteristics of the piezoelectric positioning platform. The flexible elements are made of 65 Mn, due to its excellent elasticity and fatigue strength; the remaining components are fabricated from 2A12 aluminum alloy, which is known for its high yield strength-to-Young’s modulus ratio. The piezoelectric elements are composed of PZT-4, with a density of 7.6 g/cm^3^, dimensions of 5 mm × 5 mm × 18 mm, and a Poisson’s ratio of 0.3 [40,41,42]. The finite element analysis was performed using Ansys 17.0. The mesh model was initially established via automatic meshing, while the flexure hinges were manually refined. Fixed boundary conditions were applied to the four bolt holes at the bottom of the platform. Table 1 presents the optimization results of each parameter.

**Figure 5:**
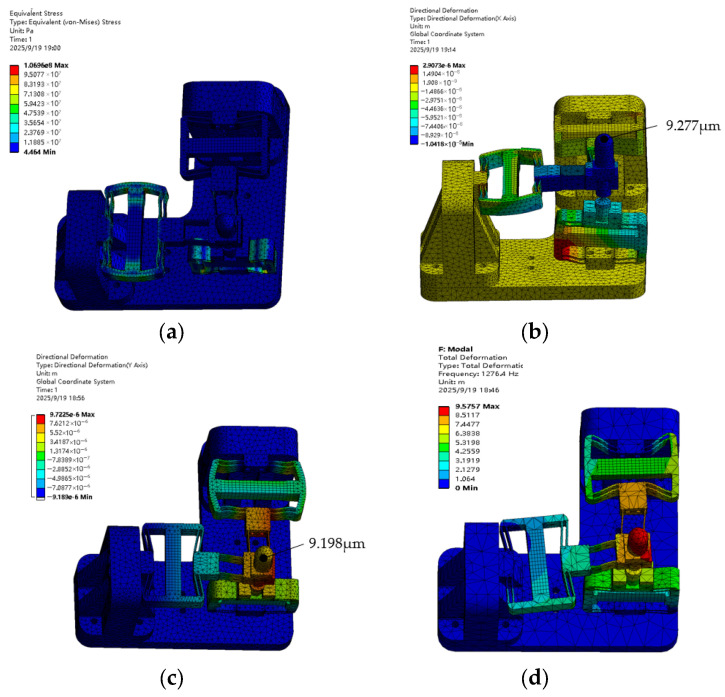
ANSYS 17.0 simulation diagram: (**a**) equivalent stress diagram; (**b**) X-direction displacement diagram; (**c**) Y-direction displacement diagram; (**d**) resonant frequency diagram.

As shown in Figure 5a, the maximum stress on the platform is 106.96 MPa, which is far lower than the material’s yield strength (310 MPa). The static simulations in Figure 5b,c indicate that the output displacement of the piezoelectric actuator is 9.277 μm in the X-direction and 9.198 μm in the Y-direction. The theoretical value is 10.12 μm for both directions, corresponding to errors of 9.08% and 10.02%, respectively. The discrepancy arises because the rectangular hinge in the orthogonal direction has finite rigidity, which reduces part of the displacement; however, the errors remain within an acceptable range. The modal analysis results in Figure 5d show that the first natural frequency obtained by simulation is 1276.4 Hz. These results theoretically confirm the validity of the proposed working principle.

## 4. Experimental Study on Inertial Platforms

### 4.1. Construction of the Experimental System

**Figure 6:**
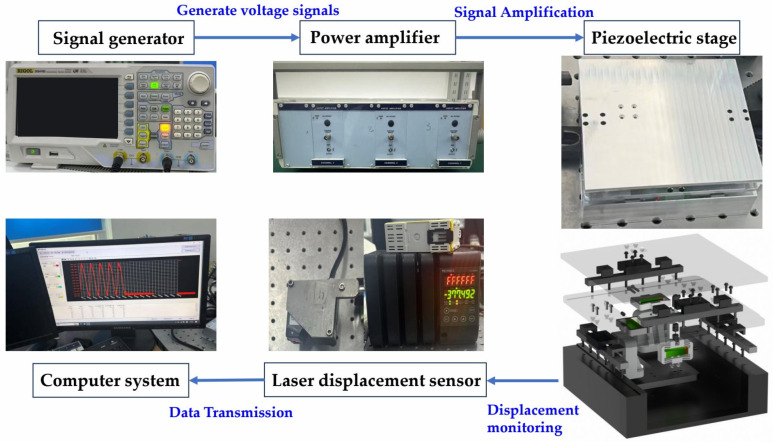
Experimental setup and prototype.

The experimental system comprises a signal generator (model DG4162; RIGOL, Beijing, China); a power amplifier with an output power of 24 W (model E00.A3; Harbin Core Tomorrow Science & Technology Co., Ltd., Harbin, China); the prototype of the proposed two-degree-of-freedom (2-DOF) piezoelectric positioning platform; a laser displacement sensor with a dynamic measurement resolution of 5 nm and a range of ±3 mm (model LK-H020; Keyence, Osaka, Japan); a computer for processing the sensor data. The signal generator is used to generate the required voltage signals; these signals are amplified by the power amplifier to drive the piezoelectric platform; the laser displacement sensors with a signal processor are applied to measure the output displacement of the piezoelectric platform. The experimental setup is shown in Figure 6.

### 4.2. Displacement Backlash in a Traditional Inertial-Drive Prototype

**Figure 7:**
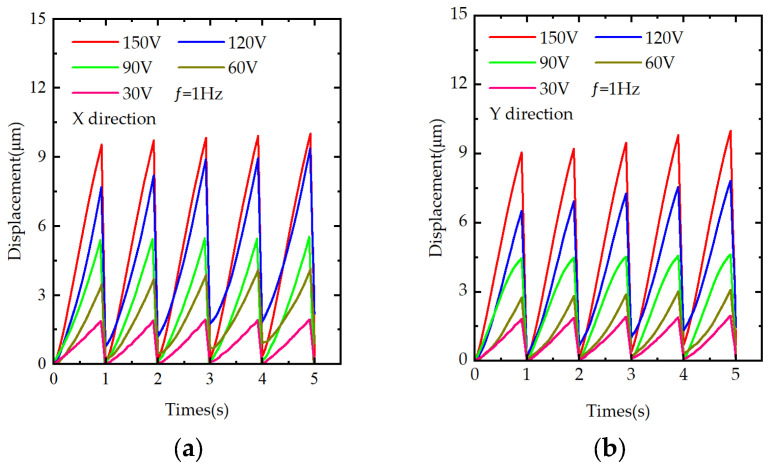
Displacement of the prototype under traditional inertial actuation: (**a**) step distance in the X-direction under different voltages; (**b**) step distance in the Y-direction under different voltages.

Experiments were performed under the traditional inertial drive, When the prototype is driven in the X and Y directions at a frequency of 1 Hz and under various driving voltages (30 V, 60 V, 90 V, 120 V, and 150 V), the displacement backlash is considerable. Specifically, the backlash rate in the X-direction ranges from 94.25% to 97.17%, while in the Y-direction, it ranges from 93.94% to 97.09%. These large backlash rates in both directions are illustrated in Figure 7a for the X-direction and Figure 7b for the Y-direction. Such high levels of displacement backlash reduce the overall precision of the prototype.

### 4.3. Fundamental Experiments on the Proposed Backlash Mitigation Model

#### 4.3.1. Displacement Experiments

**Figure 8:**
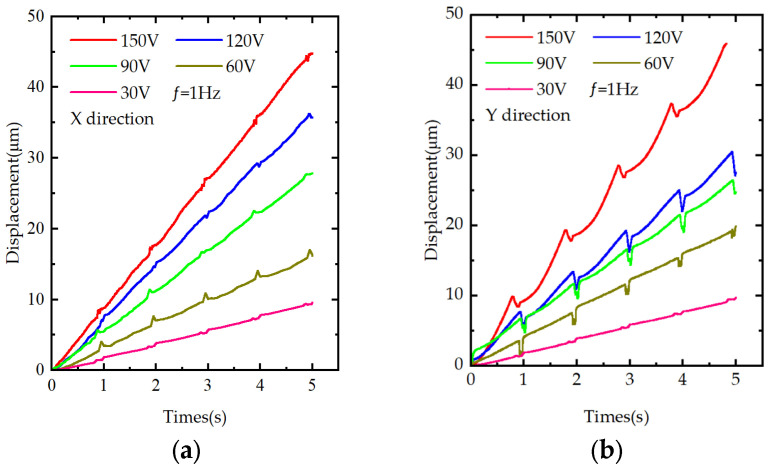
Experimental figure of step distance: (**a**) step distance in the X-direction under different voltages; (**b**) step distance in the Y-direction under different voltages.

The output displacement of the piezoelectric stack is governed by the driving voltage; therefore, the voltage strongly influences the performance of the prototype. Sawtooth-wave voltages at 1 Hz with amplitudes of 30, 60, 90, 120, and 150 V were applied to drive the prototype, and its output performance was tested under these voltages. Figure 8a,b present the resulting output displacements as the driving voltage increases from 0 to 150 V, and show the variation in motion step in the two degrees of freedom with voltage. The average single-step distances in the X-direction at 30, 60, 90, 120, and 150 V are 1.91, 3.45, 5.52, 7.28, and 8.91 μm, respectively; the corresponding values in the Y-direction are 1.94, 3.74, 4.85, 5.58, and 9.04 μm. The displacement backlash rates in the X-direction range from 0% to 9.84%, and in the Y-direction, they range from 0% to 28.42%. Under the experimental conditions, these results indicate that the proposed two-degree-of-freedom piezoelectric positioning platform eliminates the backward motion observed in inertial piezoelectric actuators operating under traditional modes in both the X and Y directions.

#### 4.3.2. Speed Experiments

**Figure 9:**
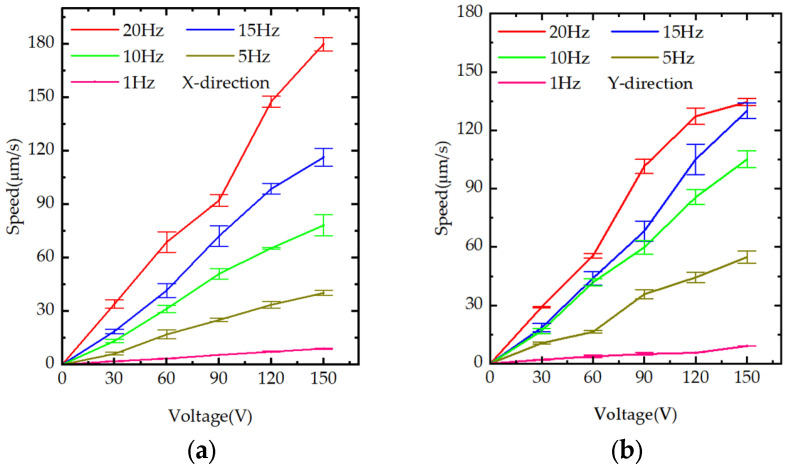
Experimental figures of speed: (**a**) speed graph in the X-direction under different frequencies and voltages; (**b**) speed graph in the Y-direction under different frequencies and voltages.

As shown in Figure 9a,b, the effects of excitation amplitude and frequency on the motion speed of the piezoelectric positioning platform were tested separately for the X and Y-directions. The excitation frequencies were set to 1, 5, 10, 15, and 20 Hz, and the voltages were set to 30, 60, 90, 120, and 150 V. By varying the excitation frequency and voltage, the motion speed was found to exhibit an approximately linear relationship with both excitation amplitude and frequency.

#### 4.3.3. Displacement Resolution Experiments

**Figure 10:**
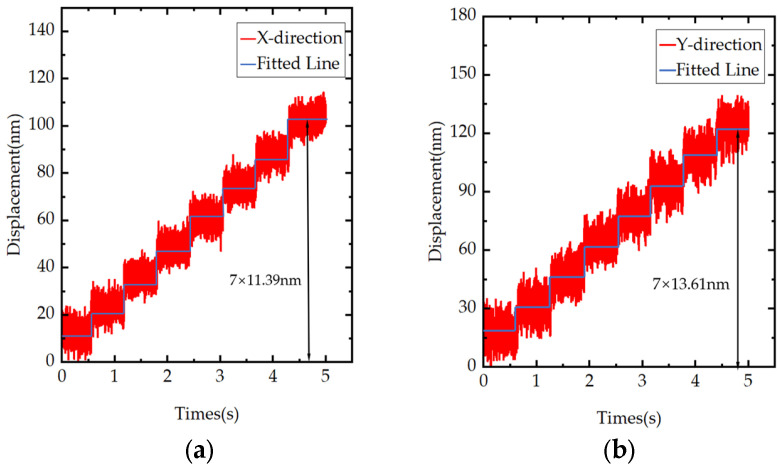
Experiments on resolutions in different directions: (**a**) X-direction resolution; (**b**) Y-direction resolution.

Resolution is a key indicator for evaluating the output performance of precision positioning platforms. A capacitive displacement sensor (model D-E20.050; Physik Instrumente GmbH & Co. KG, Karlsruhe, Germany) was used for the resolution test. This sensor has a measurement range of −25 μm to 25 μm, a static linear displacement resolution of 0.5 nm, and a dynamic linear displacement resolution of 1 nm. To reduce the influence of high-frequency noise, the sensor bandwidth was set to its minimum allowable value of 10.2 Hz during testing. The prototype’s displacement resolution was evaluated by progressively decreasing the step increment of the drive-voltage signal. When a stepwise-increasing excitation voltage with a 0.25 V increment was applied to the piezoelectric actuator, as shown in Figure 10a, the resolution in the X-direction reached 11.39 nm; as shown in Figure 10b, the resolution in the Y-direction reached 13.61 nm. These results indicate that the designed two-degree-of-freedom piezoelectric platform provides high displacement resolution and meets the design goal of high resolution.

#### 4.3.4. Resonant Frequency Experiments

**Figure 11:**
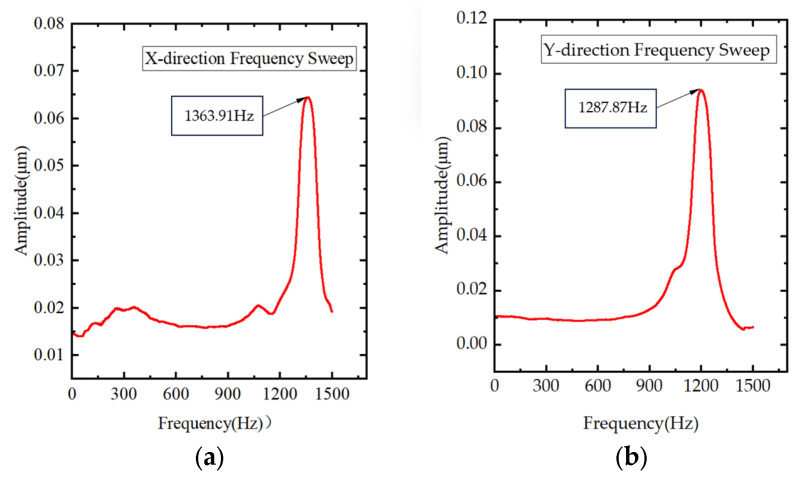
Experiments on resonant frequency in different directions: (**a**) X-direction resonant frequency; (**b**) Y-direction resonant frequency.

To clarify the dynamic performance of the designed prototype, a frequency-sweep measurement was conducted using a signal generator. The sweep voltage was 15 V and the sweep period was 10 s; the results are shown in Figure 11a,b. The measured resonant frequencies of the prototype are 1363.91 Hz in the X-direction and 1287.87 Hz in the Y-direction. For piezoelectric structures, the measured resonant frequency is commonly used to approximate the first-order resonant (natural) frequency. Therefore, the experimental results indicate strong consistency between the resonant-frequency tests and the modal analysis results.

#### 4.3.5. Trajectory Experiments

**Figure 12:**
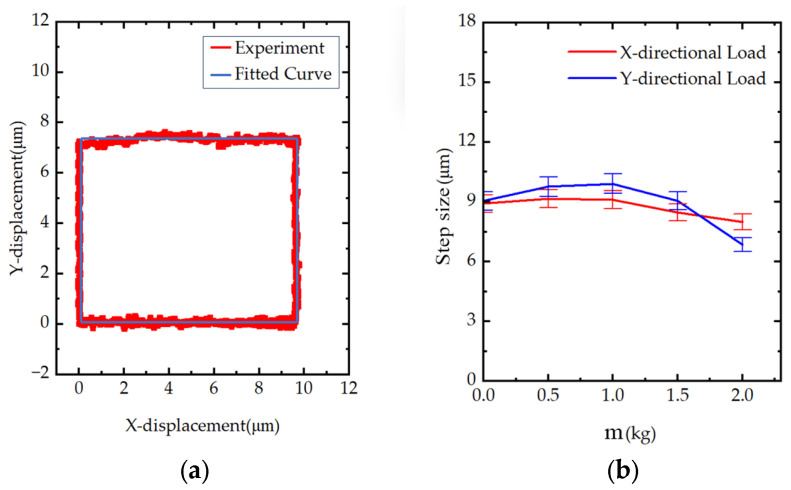
The prototype tracking and with-load experiments: (**a**) trajectory experiments; (**b**) the prototype load-carrying capacity experiments.

To verify the multi-axis coordinated motion capability of the prototype, the sampling frequency was set to 10 kHz. The experiment aimed to command the prototype to complete a closed quadrilateral trajectory in the XY plane, as shown in Figure 12a, to evaluate its trajectory-generation accuracy and motion stability. The results show that the prototype stably completed the closed motion along the quadrilateral path, with a high degree of agreement between the actual motion trajectory and the theoretical trajectory.

#### 4.3.6. The Prototype With-Load Experiments

To demonstrate the prototype’s load-carrying capacity, its motion performance was tested with weights of different masses placed on the stage. Taking the X-direction as an example, a sawtooth-wave voltage of 1 Hz and 150 V was applied to the X-direction piezoelectric actuator, and the output-displacement amplitude and backlash were measured under varying loads. The results, shown in Figure 12b, indicate that the output displacement decreases as the load increases, up to 2000 g, demonstrating good load-carrying capability.

#### 4.3.7. Application Experiments

**Figure 13:**
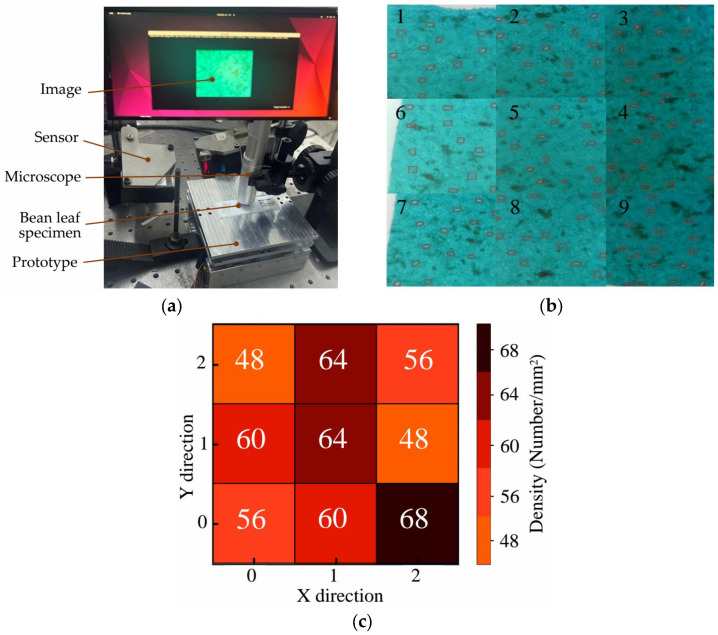
Observation experiments of broad bean leaf stomata: (**a**) observation of stomatal complex components; (**b**) stomatal observation micrograph of a broad bean leaf; (**c**) stomatal density map of broad bean leaves.

The application experiment verified the feasibility of using the prototype piezoelectric positioning prototype to observe stomatal sections of broad bean leaves. The configuration of the demonstration is illustrated in Figure 13a. To achieve array-based positioning and high-precision measurement of stomata on broad bean leaves, a microscopic measurement system was established based on this prototype. The system consists of a microscope, the prototype, a computer, and a light source.

As the core motion unit, the 2-DOF piezoelectric positioning the prototype carries the stage holding the broad bean leaf sample and provides submicron-level displacement control in the XY plane. A motion strategy was implemented: starting from the upper-left corner and moving point by point with dwells. The drive voltage applied to the prototype was 150 V at 10 Hz. The prototype first started from the upper-left corner of the observation area and moved sequentially along the X and Y axes with a preset step size, recording data every 500 μm in both directions. As shown in Figure 13b, after reaching the coordinate corresponding to the target stoma, 9 precise dwells were executed, enabled by the high stability of piezoelectric actuation; each dwell lasted 3 s to ensure the microscope captured clear stomatal images, which were obtained as shown images 1-9 in Figure 13b. In the acquired images, all stomata were marked with red rectangular boxes. The total measurement time was 117 s. The computer was used to display the microscopic images of stomata and the positioning status output by the microscope, and to record the images. The stomatal density map is shown in Figure 13c.

## 5. Conclusions

This paper designed, analyzed, and experimentally investigated a novel two-degree-of-freedom (2-DOF) piezoelectric positioning platform to address the backlash issue in inertial actuation. The structure and working principle of the proposed platform are described in detail, and its output performance is analyzed using a theoretical modeling approach. Finite element analysis (FEA) was conducted to obtain the structure’s static and dynamic characteristics, thereby verifying the accuracy of the theoretical model. Based on modeling, simulation, and experiments, the following conclusions are drawn:

1. Compared with traditional piezoelectric positioning platforms, the proposed platform employs a novel three-degree-of-freedom (3-DOF) piezoelectric stator, providing a new approach to addressing backlash in inertial actuation.
2. Experimental results show that the motion range of the platform is 15 mm × 15 mm; the displacement backlash rates in the X and Y directions range from 0% to 9.84% and 0% to 28.42%, respectively; the speeds are 177.3 μm/s and 130.4 μm/s, respectively; the displacement resolutions can reach 11.39 nm and 13.61 nm, respectively; the platform can withstand a weight of at least 2 kg or less; and the resonant frequencies in the X and Y directions are 1363.91 Hz and 1287.87 Hz, respectively.
3. The application study demonstrates the successful use of the 2-DOF platform for precision motion positioning of plant specimens under an optical microscope and for assisting in the observation of plant stomatal density, indicating its potential for botany-related microscopic observation.

In conclusion, the proposed platform exhibits high flexibility in a backlash-free inertial actuation mode and enables precise positioning and motion control at the millimeter, micrometer, and nanometer scales. It achieves accurate displacement control of plant specimens within an optical microscope system, facilitating standardized observation of plant stomatal density. This highlights its potential to improve the efficiency of microscopic feature detection and data consistency in botany, providing reliable technical support for research in plant physiology and morphology.

## Figures and Tables

**Table 1:** Material parameters of flexible hinges.

Material Parameters	Parameter Value	Material Parameters	Parameter Value
*l_a_* (mm)	5	*E* (GPa)	210
*h* (mm)	0.5	*Ρ* (kg/m^3^)	7850
*t* (mm)	1	*μ*	0.3
*d* (mm)	28	*F*_PZT_ (N)	2000

## Data Availability

The original contributions presented in the study are included in the article; further inquiries can be directed to the authors.
